# Combined truss and continuum topology optimization of structures

**DOI:** 10.1007/s00158-025-04063-2

**Published:** 2025-07-29

**Authors:** Hongjia Lu, Helen E. Fairclough, Linwei He, Matthew Gilbert

**Affiliations:** 1 Future City (Intelligent Industrial Construction) Laboratory, Innovation Center of Yangtze River Delta, Zhejiang University, Jiaxing, 314100 China; 2 School of Mechanical, Aerospace and Civil Engineering, University of Sheffield, Mappin Street, Sheffield, S1 3JD UK

**Keywords:** Continuum topology optimization, Truss topology optimization, Ground structure method, Limit analysis

## Abstract

Truss layout optimization and continuum topology optimization are both well-established methods, with each having a wide range of applications. Whereas truss layout optimization is best suited for low volume fraction problems (i.e. where the optimal structure occupies a low proportion of the original design domain), continuum topology optimization is best suited for medium and high volume fraction problems. However, real-world design problems often include both high and low volume fraction regions. To address this, a two-step hybrid optimization approach is proposed. First, low and high volume fraction regions are identified within a problem. These are then populated with truss and continuum elements respectively, which are connected via suitable interfaces. The combined optimization formulation is conic, and can be efficiently solved using interior point solvers. Numerical examples are presented to demonstrate the efficacy of the proposed approach. The results show that the approach is capable of identifying structures which contain a mixture of length scales, incorporating both bulk continuum regions and fine truss elements.

## Introduction

With the need to use high embodied carbon structural materials more sparingly in light of the climate crisis, structural optimization methods are becoming increasingly popular for a wide range of application areas.

In cases where there is significant design freedom, the minimum volume structure usually takes the form of truss-like continua (Michell 1904). However, obtaining these forms using analytical methods can be challenging, and so in practice the problem is usually considered to be one of finding the minimum volume truss containing a large, but finite, number of bars. The most common method used to obtain these optimal trusses is the ground structure method, also referred to as truss layout optimization or truss topology optimization. This approach was first proposed by Dorn et al. (1964), with computational efficiency greatly increased using the adaptive member-adding method (Gilbert and Tyas 2003). For practical purposes it is often preferable to identify structures that contain relatively few members; with the ground structure method, this is most easily achieved by using a coarse nodal grid. Whilst that restricts the possible joint positions, it can be overcome using the geometry optimization approach developed by He and Gilbert (2015).

When the structure occupies a significant proportion of the design domain, it is more appropriate to model the structure using a method that considers the more complex stress states likely to occur. Continuum-based optimization methods discretize the domain into a mesh of elements, and various methods are available to establish the presence or absence of material in each element.

The most direct formulation of this continuum approach results in a problem where the presence of material is represented by a continuous variable at each point; in two dimensions, this is interpreted as the optimization of a variable thickness sheet (Rossow and Taylor 1973). Optimal solutions to this problem can be found globally, however, the presence of intermediate material densities/thicknesses means that the solutions may not be physically applicable in many cases.

To address this, it is common to penalise the intermediate material densities (Bendsøe 1989). Various microstructure and composite layouts have been suggested as physical interpretations for these penalisation functions, however, the overall goal is usually that the optimization problem principally generates solutions where each element is either solid or void. This limits the level of detail in the structure based on the resolution used for the mesh.

Free material design (Bendsoe et al. 1994) takes an opposing approach, increasing the freedom of the design problem by allowing multiple material properties to vary independently (in the most general formulations, all components of Hooke’s tensor are independently determined). Finding practical physical interpretations of these solutions can be a significant challenge, which is usually expressed as the problem of finding microstructures that can be homogenized to the required properties (Czarnecki et al. 2017). Despite these difficulties, Lewiński et al. (2017) used a constrained version of this approach to generate an optimized lattice structure that incorporates both areas of dense and reasonably sparse structure.

Within the field of continuum optimization, it is most common to pose the problem as one of compliance minimization; i.e. to identify the stiffest layout for a given quantity of material (Bendsoe and Sigmund 2003). As a sub-category of these problems, stress constraints are sometimes imposed alongside an elastic material model to ensure that the solution remains within the elastic range (Duysinx et al. 2008).

However, many structural materials allow significant plastic capacity to be developed beyond the elastic range. Therefore, there has been interest in directly addressing the limit state of a scenario, allowing the minimization of material volume, subject to supporting a given set of loads. Lower bound formulations were considered by Kammoun and Smaoui (2014) and Kammoun (2016) for problems with single and multiple load cases, respectively. Both continuous (i.e. variable thickness sheet) and discrete (i.e. solid/void) topologies were obtained, the latter by means of a power law penalisation approach solved via a sequential conic programming approach. Conversely, Fin et al. (2019) employs a SIMP-style iterative update scheme to solve the discrete problem. A wider range of finite element types were considered by Herfelt et al. (2019), and Mourad et al. (2021) have explored the impact of alternative strength criteria. Recently, an extension to 3D problems has been proposed by Li et al. (2024). All these limit analysis approaches are capable of producing solutions with lower material usage than a comparable stress-constrained elastic approach, successfully mobilising the additional capacity available beyond the elastic range.

Various approaches have been explored with the aim of increasing understanding of the solutions produced by continuum topology optimization, including by interpreting them as truss-like structures. The moving morphable component (MMC) method proposed by Guo et al. (2014) provides an approach whereby the continuum topology optimization problem can directly provide a solution formed of discrete structural elements. This allows some of the clarity of truss optimization methods to be realized within a continuum framework. However, there are issues with the high resolution that is required to obtain solutions comparable to those which may be obtained from ground structure approaches, and the non-convexity of the problem.

Other studies have sought to interpret a truss-like structure through the post-processing of continuum optimization results. Larsen et al. (2018) extracted truss layouts from the directions of principal strains in a continuum-optimized solution, although significant post-processing was required to obtain satisfactory solutions. Notably, the centres of element ‘fans’ were explicitly identified and the boundary conditions altered to a more typical truss setup with point supports. Ma et al. (2023) automates the post-processing required, but there are still many steps involved, including a separate frame optimization to finalise the extracted design.

The wider field of de-homogenization has gained traction due to the ability to produce results with very fine length scales using relatively coarse grids, and correspondingly modest computational requirements. Interested readers are referred to Wu et al. (2021, Section5.3.1) for a review, and to Woldseth et al. (2024) for an educational example code. More recent developments have also extended the applicability to multiple load cases Jensen et al. (2022). The de-homogenized structures generally display truss-like forms in 2D cases, with larger solid regions in areas where more material is required.

Conversely, it is sometimes desirable to manufacture the structure resulting from a truss optimization from a single component; e.g. by additive manufacturing or by CNC cutting of a solid plate. It is often assumed that the joints in these cases should be thickened to prevent stress concentrations and allow for the overlapping of members converging to a single point (Smith et al. 2016). Yet, when a number of optimized trusses cut from thick plate were tested by Decker et al. (2018), it was found that the expansion of joints made little difference to the ultimate load that the structure could support, and that the generation of a plastic hinge at the non-expanded joints prevented buckling members forming more complex global modes.

Despite these challenges, it is frequently necessary in practice to combine monolithic, continuum-type structures with finer skeletal elements. A common structural application is the design of reinforced concrete. This has been the main focus of previous work on combining continuum and frame optimization. There have been several studies in which the well-known strut-and-tie approach has been guided by the results from topology optimization; a review of these approaches can be found in Xia et al. (2020). Notably, Zhong et al. (2017) proposed two methods based on a solid continuum element and a micro-truss unit respectively; for the most challenging examples, these approaches were combined in manually selected regions to generate the most appropriate strut-and-tie model.

Perhaps a more interesting method is to directly combine continuum approaches for the concrete phase and frame approaches for the reinforcement, with various formulations proposed by Amir (2013); Gaynor et al. (2013); Yang et al. (2015); Mejías and Zegard (2023). By doing this, topology optimization of concrete structure can be identified simultaneously with the reinforcement distribution within. The effectiveness of these approaches has been demonstrated experimentally by Jewett and Carstensen (2019). Later this approach was expanded to consider pre-stressed members in Amir and Shakour (2018). However, since overlapping between line and continuum elements is allowed, the application of this approach is limited to the design of reinforced concrete or other similar materials.

At a higher level, the overall design of buildings can also be considered as a combined continuum and skeletal structural design problem. Two or three-dimensional elements, most suited to continuum design approaches, may include shear walls, foundation slabs or architectural features, typically constructed from concrete. These are usually complemented by skeletal elements such as columns, beams, or transfer trusses in a wider range of materials. Zakian and Kaveh (2020) considered continuum topology optimization of shear walls, combined with a pre-defined frame structure. Optimizing the position of the shear wall within a structure is a more challenging problem, which Zhang and Mueller (2017) studied using genetic algorithms (although the impact on the surrounding frame was not considered in detail).

The aerospace and automotive sectors have, for some time now, been employing continuum optimization methods to provide high-performance and lightweight solutions for the design of individual components (Zhu et al. 2016). However, as the focus is now broadening towards the integrated design of whole vehicles, the continuum approach has become extremely computationally expensive. For example, to model a wing of an aeroplane at a reasonable resolution (voxels of up to 0.8cm dimensions), Aage et al. (2017) required up to 5 days using 8000 CPUs. Truss-based approaches have the potential to offset much of this computational difficulty. The results of Aage et al. (2017) are mostly composed of forms that could be well approximated by a frame model, even though there are also some shell or plate-like sections evident. Previous studies (e.g. Cavazzuti et al. 2011) have manually reinterpreted continuum topology optimization results into frame form, in order to improve manufacturability and avoid indistinct parts of the continuum results.

The purpose of this paper is to propose a novel approach to addresses structural topology optimization problems that contain both regions of dense and sparse structure. The approach combines truss and continuum methods in different regions of the design domain, and a simple heuristic approach is presented in order to define the division of the domain into truss and continuum areas.

The paper is structured as follows: in Sect. 2, a novel formulation with adjacent regions of skeletal and monolithic structure is described. In Sect. 3, the approach is demonstrated in an example with a pre-defined division of monolithic and skeletal structural regions. A procedure for the automatic division of the domain is then described in Sect. 4, and tested on a number of examples in Sect. 5.

## Formulations

In this section, the formulations used, both in areas of the domain solved using the truss model (Sect. 2.1) and the areas using the continuum model (Sect. 2.2), are described. Section 2.3 then proceeds to describe the interface between these two regions, allowing combined problems to be addressed.

### Truss formulation

In the truss regions of the design domain, the formulation used is the classical plastic truss layout optimization problem (Dorn et al. 1964). The region is discretized with nodes, and each pair is joined by a potential truss element to give a fully connected ground structure. The minimum volume structure is then identified through the following linear programming problem:

1a $$\begin{aligned} \min _{\textbf{q, a}} & V =\textbf{l}^\text {T}\textbf{a} \end{aligned}$$

1b $$\begin{aligned} \text {subject to } & \textbf{Bq} = \textbf{f}, \end{aligned}$$

1c $$\begin{aligned} & -\sigma _0\textbf{a} \le \textbf{q} \le \sigma _0\textbf{a}, \end{aligned}$$

1d $$\begin{aligned} & \textbf{a} \ge \textbf{0}, \end{aligned}$$

where ${\textbf{l}} = [l_1, l_2, \ldots , l_m]^\text {T}$, ${\textbf{a}} = [a_1, a_2, \ldots , a_{m}]^\text {T}$, and ${\textbf{q}} = [q_1, q_2, \ldots , q_m]^\text {T}$ denote the vectors of member lengths, cross-sectional areas, and axial forces, respectively, with *m* representing the number of members. ${\textbf{B}}$ is the equilibrium matrix consisting of direction cosines. The external force vector ${\textbf{f}} = [f_1^x, f_1^y, f_2^x, \ldots , f_n^y]^\text {T}$ consists of the external forces at *n* nodes, with $f_j^x$ and $f_j^y$ indicating the external forces at node *j* in the *x* and *y* directions, respectively. Where a degree of freedom is supported (i.e. the external/reaction force *f* can be freely chosen) then equilibrium will always be satisfied and the corresponding row of the constraint is removed from the problem. Finally, $\sigma _0$ denotes the ultimate stress of the material.

### Continuum formulation

**Fig. 1:**
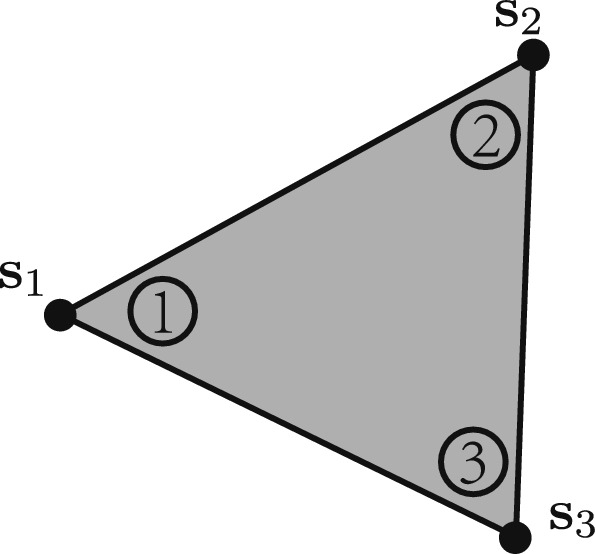
Stress components of a continuum element, showing the corners 1, 2, 3, at which the variables representing stress components, ${\textbf{s}}_1$, ${\textbf{s}}_2$, ${\textbf{s}}_3$, are defined. Within the element, the stresses have a linear distribution between these values

The continuum approach used in this study generally follows Kammoun and Smaoui (2014). For the sake of completeness, the optimization formulation is reviewed in this section.

In the continuum region, a lower bound formulation using triangular mesh elements is employed. In each element, stresses are permitted to vary linearly and optimization variables representing the components of the stress tensor at each corner of the element are required, $\sigma _x, \sigma _y$ and $\tau _{xy}$ will denote the normal stresses in the x and y directions and the shear stress, respectively. For convenience, these will be represented within the stress tensor at a location *i*, ${\textbf{s}}_i = [\sigma _{x,i}, \sigma _{y,i}, \tau _{xy, i}]^\text {T}$. Note that although the corners of multiple elements may meet at a single point, these different corners will have independent stress variables (Kammoun and Smaoui 2014). In this paper, the term corner will be used to describe these locations within the continuum part of the structure, whilst the term node will be reserved for the usual definition within truss layout optimization.

For the arbitrary element, *e*, as shown in Fig. 1, the internal force equilibrium is given by:

2 $$\begin{aligned} {\textbf{N}}_e{\varvec{\sigma }}_e = {\textbf{0}}, \end{aligned}$$

where ${\textbf{N}}_e$ is a matrix containing shape functions of element *e*, and ${\varvec{\sigma }}_e = [{\textbf{s}}_1^\text {T}, {\textbf{s}}_2^\text {T}, {\textbf{s}}_3^\text {T}]^\text {T}$ is a stress vector containing the stresses at the three corners of *e*, as shown in Fig. 1. Detailed values of ${\textbf{N}}_e$ and ${\varvec{\sigma }}_e$ can be found in the appendix.

**Fig. 2:**
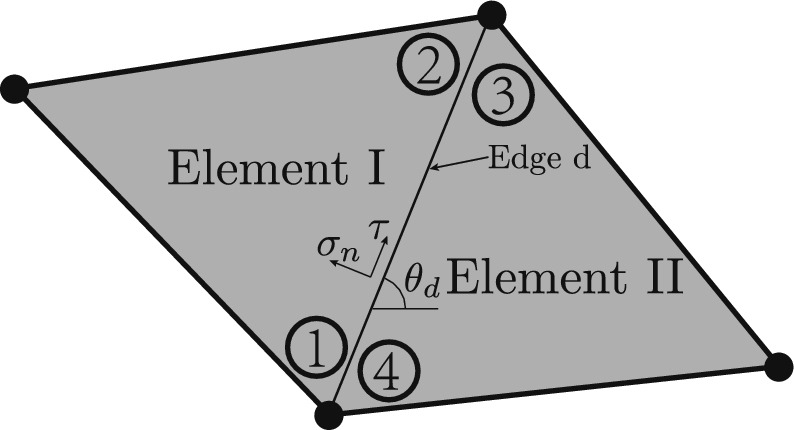
Force equilibrium between two elements at a common edge, with $\theta _d$, $\sigma _n$ and $\tau$ representing the edge orientation angle, the normal stress to the edge, and the shear stress along the edge, respectively

Along the edge joining two adjacent elements, statically admissible discontinuities in the stress field are permitted, thus the required constraints enforce the continuity of normal and shear stresses along the edge. As the stress field is linear within each element, these constraints need only be applied at the ends of the edge. Therefore, for an arbitrary pair of elements sharing a common edge, *d*, as shown in Fig. 2, the force equilibrium at the edge is given by

3 $$\begin{aligned} {\textbf{A}}_d {\varvec{\sigma }}_{d,\textrm{I}} = {\textbf{A}}_d {\varvec{\sigma }}_{d,\mathrm{I\hspace{-0.2ex}I}}, \end{aligned}$$

where ${\textbf{A}}_{d}$ is a transformation matrix containing sine and cosine values of the edge angle $\theta _d$, and ${\varvec{\sigma }}_{d,\textrm{I}}$ and ${\varvec{\sigma }}_{d,\mathrm{I\hspace{-0.2ex}I}}$ are stress vectors containing the stresses at relevant corners in element I (corners 1 and 2 in Fig. 2) and II (corners 3 and 4 in Fig. 2). Detailed values of ${\textbf{A}}_{d}$, ${\varvec{\sigma }}_{d,\textrm{I}}$ and ${\varvec{\sigma }}_{d,\mathrm{I\hspace{-0.2ex}I}}$ can be found in the appendix.

**Fig. 3:**
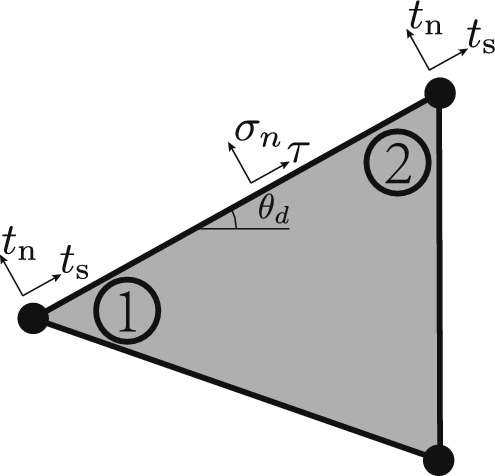
Force equilibrium at a domain boundary edge, where $t_\text {n}$ and $t_\text {s}$ represent the normal stress and shear stress, respectively. The applied stress is user-defined individually for each corner. Along each edge the applied stress varies linearly to give $\sigma _n$ and $\tau$, the intermediate normal stress to the edge, and the intermediate shear stress along the edge, respectively. $\theta _d$ represents the edge orientation angle

For boundary edges, the stresses must equal zero unless the edge is applied with external force. For an arbitrary element with an edge located at the domain boundary, as shown in Fig. 3, the force equilibrium at the boundary edge is shown in (4):

4 $$\begin{aligned} {\textbf{A}}_{d}{\varvec{\sigma }}_{d} = {\textbf{t}}_d, \end{aligned}$$

where ${\varvec{\sigma }}_d$ is a stress vector that contains the stresses at corners 1, 2 in Fig. 3, and the external load vector, ${\textbf{t}}_d$, contains the stresses applied at corner 1 and 2 in Fig. 3. Note that, due to the linear variation of the stress field within each element, defining the stresses at the end-points is sufficient to define the applied stress distribution along the entire edge, see Kammoun and Smaoui (2014) for more information. Detailed values for ${\varvec{\sigma }}_d$ and ${\textbf{t}}_d$ can be found in the appendix.

The von Mises yield criterion is enforced at each corner of each mesh element, with the maximum von Mises stress linearly scaled according to the density (or depth, when considered as a variable thickness sheet problem) at that corner. This gives a conic constraint, which at each corner can be written as:

5 $$\begin{aligned} \sigma _{x}^2 + \sigma _{y}^2 + \left( \sigma _{x}-\sigma _{y}\right) ^2 + \left( \sqrt{6}\tau _{xy}\right) ^2 \le \left( \sqrt{2}\sigma _0 \rho \right) ^2, \end{aligned}$$

where $\rho$ is the density at the relevant corner point, $\sigma _0$ is the ultimate stress of the material and the remaining stress variables are as previously defined.

Constraint (5) can be posed as a quadratic conic constraint, ensuring that solutions obtained are globally optimal and allowing the use of modern, computationally efficient solvers.

Similar to the truss optimization problem, the objective of the continuum optimization problem is to minimize the total volume that is equal to ${\textbf{v}}^\text {T}\varvec{\rho }$, where ${\textbf{v}}$ is the volume vector of the elements, ${\textbf{v}} = \frac{1}{3}[A_1, A_1, A_1, A_2,..., A_{{\bar{n}}}]^\textrm{T}$. $A_e$ is the area of element *e*, and $\varvec{\rho }$ is the vector containing the density variables $\rho$ for each corner of each element.

To sum up, the continuum optimization formulation is shown in Eqs. (6). The set ${\mathbb {C}}$ contains all continuum elements, whilst set ${\mathbb {P}}$ contains all corners of these elements (i.e. set ${\mathbb {P}}$ is 3 times the size of ${\mathbb {C}}$). Note that, unlike in most compliance-based formulations, here adjacent elements are connected by their edges, rather than by common nodes. The internal edges of the mesh are contained in set ${\mathbb {E}}$, whilst unsupported boundary edges are contained in set ${\mathbb {B}}$. Finally, supported edges are contained in set ${\mathbb {O}}$. On supported edges, equilibrium is automatically satisfied (c.f. the truss formulation above) thus set ${\mathbb {O}}$ does not explicitly appear in any of the equilibrium constraints in (6).

6a $$\begin{aligned} \min _{\varvec{\rho }, {\varvec{\sigma }}}\hspace{3pt}&\textbf{v}^\textrm{T} \varvec{\rho }&\text { Total volume } \end{aligned}$$

6b $$\begin{aligned} \text {subject to }&\textbf{N}_{e} {\varvec{\sigma }}_e = \textbf{0}, \; \forall e \in \mathbb {C}&\text {Continuum element equilibrium} \end{aligned}$$

6c $$\begin{aligned}&\textbf{A}_d {\varvec{\sigma }}_{d,\textrm{I}} - \textbf{A}_d {\varvec{\sigma }}_{d,\mathrm{I\hspace{-0.2ex}I}} = \textbf{0}, \;\forall d \in \mathbb {E}&\text {Continuum edge equilibrium} \end{aligned}$$

6d $$\begin{aligned}&\textbf{A}_d {\varvec{\sigma }}_d = \textbf{t}_d, \;\forall d \in \mathbb {B}&\text {Continuum boundary equilibrium} \end{aligned}$$

6e $$\begin{aligned}&\sigma _{x,j}^2 + \sigma _{y,j}^2 + \left( \sigma _{x,j}-\sigma _{y,j}\right) ^2 + \left( \sqrt{6}\tau _{xy,j}\right) ^2 \le \left( \sqrt{2}\sigma _0 \rho _j\right) ^2, \forall j \in \mathbb {P}\,\,\,\,\,\text {Continuum VM yield} \end{aligned}$$

### Interfaces

This section discusses how truss and continuum formulations can be combined to form a single problem. For the purposes of this paper, it is assumed that truss elements connect to continuum elements only along a pre-defined set of edges that lie on the boundary of the continuum region.

#### One-to-many interface

**Fig. 4:**
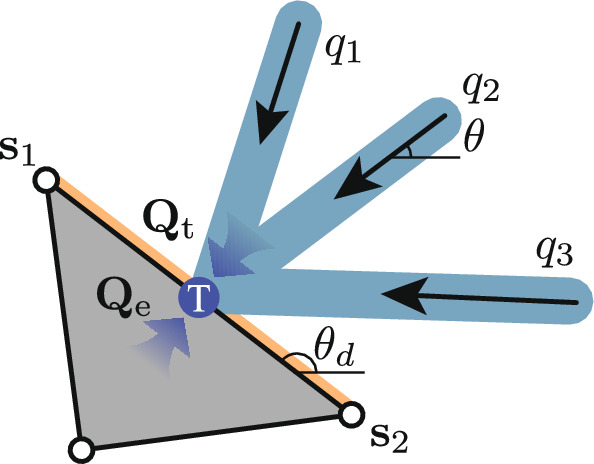
The interface T between a continuum element and three truss elements, where ${\textbf{s}}_1$ and ${\textbf{s}}_2$ represent the nodal stress vectors for the continuum element, and *q* denotes the axial force from the truss elements; $\theta _d$ is the orientation angle of the interface edge; ${\textbf{Q}}_t$ and ${\textbf{Q}}_e$ are the resultant force vectors for the truss elements and the continuum element, respectively

This section describes the linkage between the continuum and truss regions. As illustrated in Fig. 4, a number of truss elements can connect to a single edge of a continuum element. Due to the way external forces are applied in the continuum formulation, truss elements must connect to edges of continuum elements, rather than, e.g. the corner-points of the continuum discretization. It is most convenient to locate the end-point of the truss element at the midpoint of the relevant continuum edge. When constructing a hybrid problem, this will be ensured by generating additional truss nodes as required along the edges of the continuum regions.

At the interface, the resultant forces ${\textbf{Q}}_\text {e}$ integrated along the edge should be equal to the resultant forces ${\textbf{Q}}_\text {t}$ from the truss elements. Thus for an edge *d* connecting corners 1 and 2 of the continuum element (e.g. Figure 4), the required constraint is

7 $$\begin{aligned} {\textbf{Q}}_\text {e} + {\textbf{Q}}_\text {t} = \frac{l_d}{2}\begin{bmatrix}{\textbf{U}}_d&{\textbf{U}}_d \end{bmatrix} \begin{bmatrix}{\textbf{s}}_\text {1} \\ {\textbf{s}}_\text {2} \end{bmatrix} + {\textbf{B}}_d{\textbf{q}} = \begin{bmatrix}0 \\ 0 \end{bmatrix}, \end{aligned}$$

where $l_d$ is the length of edge *d*, and ${\textbf{s}}_i = [\sigma _{x,i}, \sigma _{y,i}, \tau _{xy, i}]^\text {T}$ for a corner *i*; i.e. $[{\textbf{s}}_1, {\textbf{s}}_2]^\textrm{T} = {\varvec{\sigma }}_d$. Finally, ${\textbf{B}}_d$ is a sub-matrix of ${\textbf{B}}$ containing only the rows corresponding to the node located at the centre of edge *d*, and

8 $$\begin{aligned} {\textbf{U}}_d = \begin{bmatrix} -\sin \theta _d & 0 & \cos \theta _d \\ 0 & \cos \theta _d & -\sin \theta _d \end{bmatrix}, \end{aligned}$$

with $\theta _d$ the angle between the edge and the positive *x*-axis. Note that this constraint replaces the rows of the truss equilibrium constraint relevant to the node in the centre of edge *d*, and also replaces the continuum boundary constraints on edge *d*.

As Eq. (7) only constrain the integral of the stress along the element, an additional constraint is required to ensure moment equilibrium (to prevent cases where, e.g. $\Vert {\textbf{s}}_1\Vert \gg \Vert {\textbf{s}}_2\Vert = 0$ in Fig. 4). The simplest formulation of this is to take moments about the centre of the edge:

9 $$\begin{aligned} \begin{bmatrix}{\textbf{M}}_d&-{\textbf{M}}_d \end{bmatrix} \begin{bmatrix} {\textbf{s}}_1 \\ {\textbf{s}}_2 \end{bmatrix} = 0, \end{aligned}$$

where $${\textbf{M}}_d = [\sin ^2\theta _d, \cos ^2\theta _d, -2\cos \theta _d\sin \theta _i]$$.

There are now four sets of edge types in the continuum problem that need to be distinguished:

- As previously, set ${\mathbb {E}}$ contains the continuum edges that are internal to the continuum region,
- Set ${\mathbb {O}}$ contains supported edges (and does not explicitly appear in the final formulation).
- Set ${\mathbb {B}}$ contains edges that lie on the boundary of the continuum region, excluding those that are on the interface or that are supported.
- Set ${\mathbb {I}}$ contains continuum edges that lie on the boundary with the truss region.

For each edge *d* in ${\mathbb {I}}$, ${\textbf{B}}_{d}$ is also defined, which is the row of ${\textbf{B}}$ corresponding to the truss node located on edge *d*. In addition, ${\textbf{B}}_\textrm{t}$ is defined, which is the remaining rows of ${\textbf{B}}$ once all the rows corresponding to nodes on the interface are removed. The external (truss) force vector ${\textbf{f}}$ is similarly split into ${\textbf{f}}_\textrm{t}$ and ${\textbf{f}}_{d} \forall d \in {\mathbb {I}}$. The full hybrid formulation with one-to-many interfaces is shown in (10).

10a $$\begin{aligned} \min _{\varvec{\rho }, {\varvec{\sigma }}, \textbf{a}, \textbf{q}}&\textbf{l}^\textrm{T} \textbf{a} + \textbf{v}^\textrm{T} \varvec{\rho }&\text { Total volume } \end{aligned}$$

10b $$\begin{aligned} \text {subject to }&\textbf{B}_t\textbf{q} = \textbf{f}_t&\text { Truss equilibrium} \end{aligned}$$

10c $$\begin{aligned}&\textbf{N}_e {\varvec{\sigma }}_e = \textbf{0}, \;\forall e \in \mathbb {C}&\text {Continuum element equilibrium} \end{aligned}$$

10d $$\begin{aligned}&\textbf{A}_d {\varvec{\sigma }}_{d,\textrm{I}} - \textbf{A}_d {\varvec{\sigma }}_{d,\mathrm{I\hspace{-0.2ex}I}} = \textbf{0},\;\forall d \in \mathbb {E}&\text {Continuum edge equilibrium} \end{aligned}$$

10e $$\begin{aligned}&\textbf{A}_d {\varvec{\sigma }}_d = \textbf{t}_d, \;\forall d \in \mathbb {B}&\text {Continuum boundary equilibrium} \end{aligned}$$

10f $$\begin{aligned}&\textbf{B}_{d}\textbf{q} + \frac{l_d}{2} \begin{bmatrix} \textbf{U}_d&\textbf{U}_d \end{bmatrix} {\varvec{\sigma }}_d = \textbf{f}_d,\; \forall d \in \mathbb {I}&\text {Interface equilibrium} \end{aligned}$$

10g $$\begin{aligned}&\begin{bmatrix}\textbf{M}_d&-\textbf{M}_d\end{bmatrix} {\varvec{\sigma }}_d = 0, \;\forall d \in \mathbb {I}&\text {Interface moment equilibrium} \end{aligned}$$

10h $$\begin{aligned}&-\sigma _0\textbf{a} \le \textbf{q} \le \sigma _0\textbf{a}&\text {Truss yield} \end{aligned}$$

10i $$\begin{aligned}&\sigma _{x,j}^2 + \sigma _{y,j}^2 + \left( \sigma _{x,j}-\sigma _{y,j}\right) ^2 + \left( \sqrt{6}\tau _{xy,j}\right) ^2 \le \left( \sqrt{2}\sigma _0 \rho _j\right) ^2, \; \forall j\in \mathbb {P}&\,\,\,\,\,\text {Continuum VM yield} \end{aligned}$$

#### Many-to-many interface

In some cases, the force carried by a truss bar may be larger than the force that can be carried by a single element in the continuum region. Assuming that the continuum and truss areas are constructed from materials with similar permitted stresses, this implies that the cross-section of the truss bar will be larger than the edge of a single continuum element, and will be in contact with at least part of the adjacent elements. Therefore, the formulation is now extended to allow truss bars that connect to multiple continuum elements.

**Fig. 5:**
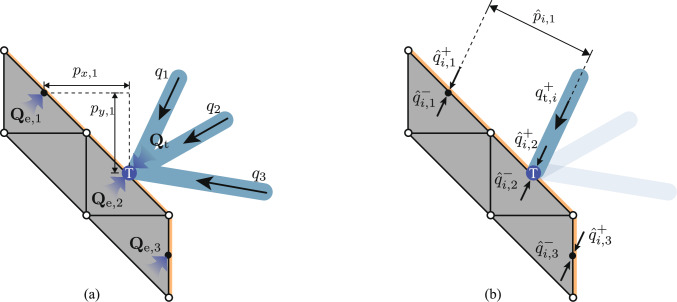
The interface T between three truss elements and three continuum elements: **a** global equilibrium between the resultant force vectors ${\textbf{Q}}_\text {t}$ of the truss elements and ${\textbf{Q}}_{\text {e}, 1}$ to ${\textbf{Q}}_{\text {e}, 3}$ of the continuum elements. This is comparable to the approach in Sect. 2.3.1; **b** localized equilibrium between one truss element, *i*, and three continuum elements, where ${\hat{q}}^+_{{i,d}}$ and ${\hat{q}}^-_{{i,d}}$ represent the positive and negative forces on the *d*th edge, parallel to the concerned truss element *i*; $q^+_{\text {t},i}$ denotes the positive axial force of the concerned truss element. This is the approach required in Sect. 2.3.2

Figure 5a shows one end of three truss elements and three continuum edges to which they may connect. In this work, each truss element is able to connect to continuum edges whose centre points lie within a given distance of the truss node which is specified as part of the problem description. For each truss node, this therefore defines the set ${\mathbb {J}}_{i} (\subset {\mathbb {I}})$ of edges to which connections are possible. Note that the end-point of the truss element is no longer required to be co-incident to the centre of any continuum edge (but for the purposes of comparability, in this paper they usually are).

In this situation, the force and moment equilibrium equations at the interface would be:

11 $$\begin{aligned} & {\textbf{Q}}_\text {t} + \sum _{\forall d \in {\mathbb {J}}_i} {\textbf{Q}}_{\text {e}, d} = \begin{bmatrix}0 \\ 0 \end{bmatrix}, \end{aligned}$$

12 $$\begin{aligned} & \sum _{\forall d \in {\mathbb {J}}_i} {\textbf{Q}}_{\text {e}, d}^\text {T} \begin{bmatrix} p_{x, d} \\ p_{y, d} \end{bmatrix} = 0, \end{aligned}$$

where ${\textbf{Q}}_{\text {e}, d}$ represent the resultant force vectors of the *d*th element edge; $p_{x,d}$ and $p_{y,d}$ denote x- and y-distances, respectively, between the centre point of the *d*th edge and the interface node (see, e.g., Fig. 5a).

As the force in the bar can be split into many components, it is necessary to ensure that the optimizer cannot use a combination of positive and negative forces which is not physically valid. An example of this would be ${\textbf{Q}}_\text {t} = [0, 0]^\text {T}$, ${\textbf{Q}}_{\text {e}, 1} = [0, 1]^\text {T}$, ${\textbf{Q}}_{\text {e}, 2} = [0, -2]^\text {T}$ and ${\textbf{Q}}_{\text {e}, 3} = [0, 1]^\text {T}$ in Fig. 5a, which satisfies constraints (11) and (12) but is not valid in the practical sense.

To address this issue, it is convenient to make use of non-negative optimization variables, with the tensile and compressive forces separated (the direction of each component indicated by the superscript $+$ or −). Here it also becomes convenient to consider separate components for each truss element connecting at the node, rather than the overall resultant of all truss forces. The components, denoted as ${\hat{q}}^\pm _{i, d}$, are defined at each edge, *d*, that bar-end *i*, is permitted to connect to. Also, at the truss node itself $q_{\text {t},i}^\pm$ components allow connected truss elements to directly transfer forces between themselves if necessary, using a standard truss equilibrium constraint. All of these components are defined to be in line with the centre-line of the truss element, as shown in Fig.  5b. The equilibrium of the end plate in the direction perpendicular to the centre-line of the bar relevant to *i* is therefore trivially satisfied, as no forces act in this direction. Equilibrium parallel to the centre-line for the end-point of a bar-end *i* is enforced with the following constraint:

13 $$\begin{aligned} q_i + q_{\text {t},i}^+ + \sum _{\forall {d} \in {\mathbb {J}}_{i}}{\hat{q}}^+_{i,d} = q_{\text {t}, i}^- + \sum _{\forall {d} \in {\mathbb {J}}_{i}} {\hat{q}}^-_{i,d}, \end{aligned}$$

where the set ${\mathbb {J}}_{i}$ contains all edges to which bar-end *i* is permitted to connect.

Moment equilibrium of the end plate is enforced by taking moments about the truss node to give

14 $$\begin{aligned} \sum _{\forall {d} \in {\mathbb {J}}_{i}} \hat{p}_{{i,d}} \left( {\hat{q}}_{{i,d}}^+ - {\hat{q}}_{{i,d}}^-\right) = 0, \end{aligned}$$

where $\hat{p}_{{i,d}}$ represents the distance perpendicular to truss bar *i* from the interface node T to the centre of the *d*th element edge, as shown in Fig. 5b. Note that $\hat{p}_{i,d}$ varies in sign depending on which side of the bar the centre point lies on.

With this approach, the invalid combination of positive and negative forces can be avoided by ensuring that the total magnitude of the component forces does not exceed the load carrying capacity of the truss element. For this, the following constraint will be used:

15 $$\begin{aligned} \sum _{\forall {d} \in {\mathbb {J}}_{i}} ({\hat{q}}_{i, {d}}^+ + {\hat{q}}_{i, {d}}^-) \le \sigma _0 a_i. \end{aligned}$$

**Fig. 6:**
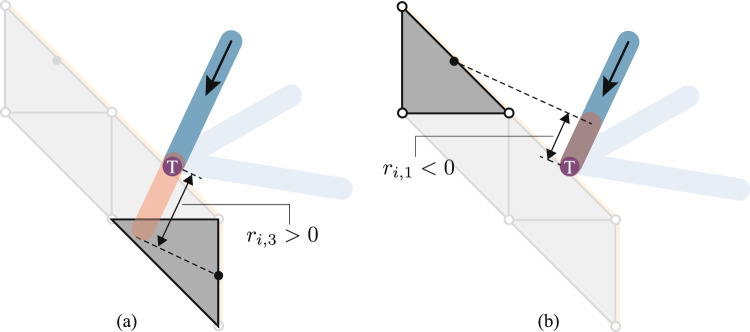
Truss element length adjustment to compensate for misalignment between the interface node T and the concerned edge: **a** a positive value of $r_{i,3} > 0$ is required between the highlighted truss element and continuum element 3; **b** a negative value of $r_{i,1} < 0$ is required between the highlighted truss element and continuum element 1

For the many-to-many interface, ideally, the components closest to the truss end-point should be used first. To achieve this, the edges are first sorted based on ${\hat{p}}_{i,d}$, to give the ordered set $\dot{{\mathbb {J}}}_{i}$ (note that this order may vary between different truss elements connected at the same node). The following constraints are then defined between each consecutive pair of edges drawn from $\dot{{\mathbb {J}}}_{i}$:

16 $$\begin{aligned} \text {sgn}({\hat{p}}_{i,d} + {\hat{p}}_{i,d+1}) \begin{bmatrix} {\hat{q}}_{i, d}^+ - {\hat{q}}_{i, d+1}^+ \\ {\hat{q}}_{i, d}^- - {\hat{q}}_{i, d+1}^- \end{bmatrix} \le \begin{bmatrix} 0 \\ 0 \end{bmatrix}, \forall d, d+1 \in \dot{{\mathbb {J}}}_{i} \end{aligned}$$

where *d* denotes the edge index; the use of the sign function, sgn, allows the direction of inequality to be effectively reversed for elements with negative ${\hat{p}}_{i,d}$. There are therefore 4 independent chains of inequalities for each truss element.

Finally, when calculating the volume of the truss element, the element length is adjusted to account for the misalignment between the interface node and the corresponding element edge, as shown in Fig. 6. Specifically, in Fig. 6a, the misalignment between the interface node T and the edge centre point requires the truss bar segment connecting them to be extended, parallel to the centreline, beyond the truss end-point, thereby increasing the length of that portion of the truss bar. In contrast, the reverse adjustment may apply in the situation depicted in Fig. 6(b). To compensate for this, the parallel distance from the truss element’s end-point to the continuum edge’s midpoint, denoted as $r_{i,d}$, is used. The value of $r_{i,d}$ may be either positive or negative. Subsequently, terms of the form $r_{i,d} ({\hat{q}}_{i,d}^+ + {\hat{q}}_{i,d}^-)$ are added to the objective function for each edge.

Equations (17) show the full formulation for the problem with many-to-many linkage, where the set of edges that may connect to bar-end *i* is given by ${\mathbb {J}}_i$. For the purposes of this paper, set ${\mathbb {J}}_i$ is defined by a certain distance from the bar-end to the edge centre, which is specified in the problem description. The set of all bar-ends on the interface is defined as ${\mathbb {D}}$, whilst set ${\mathbb {T}}$ contains the truss nodes on the interface (each with multiple bar-ends from ${\mathbb {D}}$ attached). The vectors $\hat{{\textbf{q}}}^\pm _d$ collect all variables ${\hat{q}}^\pm _{i,d}$ for each bar-end *i* which is permitted to connect to edge *d*. Meanwhile, ${\textbf{q}}^\pm _{\text {t}, j}$ collects the variables $q_{\text {t},i}^\pm$ for each bar-end *i* connected to (truss) node *j*.

17a $$\begin{aligned} \min _{\varvec{\rho }, {\varvec{\sigma }}, \textbf{a}, \textbf{q}}&\textbf{l}^\textrm{T} \textbf{a} + \textbf{v}^\textrm{T} \varvec{\rho }+ \sum _{\forall i}\sum _{\forall {d} \in \mathbb {J}_i} r_{{i},{d}}(\hat{q}^+_{i,{d}} + \hat{q}^-_{i,{d}})&\text { Total volume } \end{aligned}$$

17b $$\begin{aligned} \text {subject to }&\textbf{B}_\text {t}\textbf{q} = \textbf{f}_t&\text {Truss equilibrium} \end{aligned}$$

17c $$\begin{aligned}&\textbf{B}_j(\textbf{q}^+_{\text {t}, j} - \textbf{q}^-_{\text {t}, j}) = \textbf{f}_j, \forall j \in \mathbb {T},&\text {Truss Equilibrium (interfaces)} \end{aligned}$$

17d $$\begin{aligned}&\textbf{N}_e {\varvec{\sigma }}_e = \textbf{0},\;\forall e \in \mathbb {C}&\text {Continuum element equilibrium} \end{aligned}$$

17e $$\begin{aligned}&\textbf{A}_d {\varvec{\sigma }}_{d,\textrm{I}} - \textbf{A}_d {\varvec{\sigma }}_{d,\mathrm{I\hspace{-0.2ex}I}} = \textbf{0},\;\forall d \in \mathbb {E}&\text {Continuum edge equilibrium} \end{aligned}$$

17f $$\begin{aligned}&\textbf{A}_d {\varvec{\sigma }}_d = \textbf{t}_d,\;\forall d \in \mathbb {B}&\text {Continuum boundary equilibrium} \end{aligned}$$

17g $$\begin{aligned}&\textbf{B}_{d}(\hat{\textbf{q}}_d^+ - \hat{\textbf{q}}_d^-) + \frac{l_i}{2}\begin{bmatrix} \textbf{U}_d&\textbf{U}_d \end{bmatrix} {\varvec{\sigma }}_d = \textbf{0}, \\&\quad \forall d \in \mathbb {I}\;\;\;\text {Interface edge equilibrium} \end{aligned}$$

17h $$\begin{aligned}&\frac{l_d}{2} \begin{bmatrix}\textbf{M}_d&-\textbf{M}_d\end{bmatrix} {\varvec{\sigma }}_d = 0,\\& \forall d \in \mathbb {I}\,\text { Moment equilibrium of interface edge} \end{aligned}$$

17i $$\begin{aligned}&\ q_i + q_{\textrm{t},i}^+ - q_{\textrm{t},i}^- + \sum _{\forall {d} \in \mathbb {J}_i}(\hat{q}^+_{i,d} - \hat{q}^-_{i,d}) =0,\\ &\forall i \in \mathbb {D}\,\text {Interface in-line equilibrium} \end{aligned}$$

17j $$\begin{aligned}&\sum _{\forall d \in \mathbb {J}_i} \hat{p}_{i,d} (\hat{q}_{i,d}^+ - \hat{q}_{i,d}^-) = 0,\\& \forall i \in \mathbb {D}&\text {Moment equilibrium of truss end-point} \end{aligned}$$

17k $$\begin{aligned}&\sigma _0 a_i - \sum _{\forall d \in \mathbb {J}_i} (\hat{q}_{i,d}^+ + \hat{q}_{i,d}^-) \ge 0,\\&\forall i \in \mathbb {D}\,\text {Interface capacity} \end{aligned}$$

17l $$\begin{aligned}&\text {sgn}(\hat{p}_{i, d} + \hat{p}_{i,d+1}) \begin{bmatrix} \hat{q}_{i,d}^+ - \hat{q}_{i,d+1}^+ \\ \hat{q}_{i,d}^- - \hat{q}_{i,d+1}^- \end{bmatrix} \le \begin{bmatrix} 0 \\ 0 \end{bmatrix}, \\&\forall {d, d}+1 \in \dot{\mathbb {J}}_i,\, \forall i \in \mathbb {D}\hspace{-10pt}\,\text {Closest connections first} \end{aligned}$$

17m $$\begin{aligned}&-\sigma _0\textbf{a} \le \textbf{q} \le \sigma _0\textbf{a}&\text {Truss yield} \end{aligned}$$

17n $$\begin{aligned}&\sigma _{x,j}^2 + \sigma _{y,j}^2 + \left( \sigma _{x,j}-\sigma _{y,j}\right) ^2 + \left( \sqrt{6}\tau _{xy,j}\right) ^2\\& \le \left( \sqrt{2}\sigma _0 \rho _j\right) ^2 ,\;\forall j\in \mathbb {P}\,\text {Continuum VM yield} \end{aligned}$$

17o $$\begin{aligned}&\hat{\textbf{q}}^+, \hat{\textbf{q}}^-, \textbf{q}^+_\text {t}, \textbf{q}^-_\text {t} \ge \textbf{0} \end{aligned}$$

## Example with fixed regions of truss and continuum domain

**Fig. 7:**
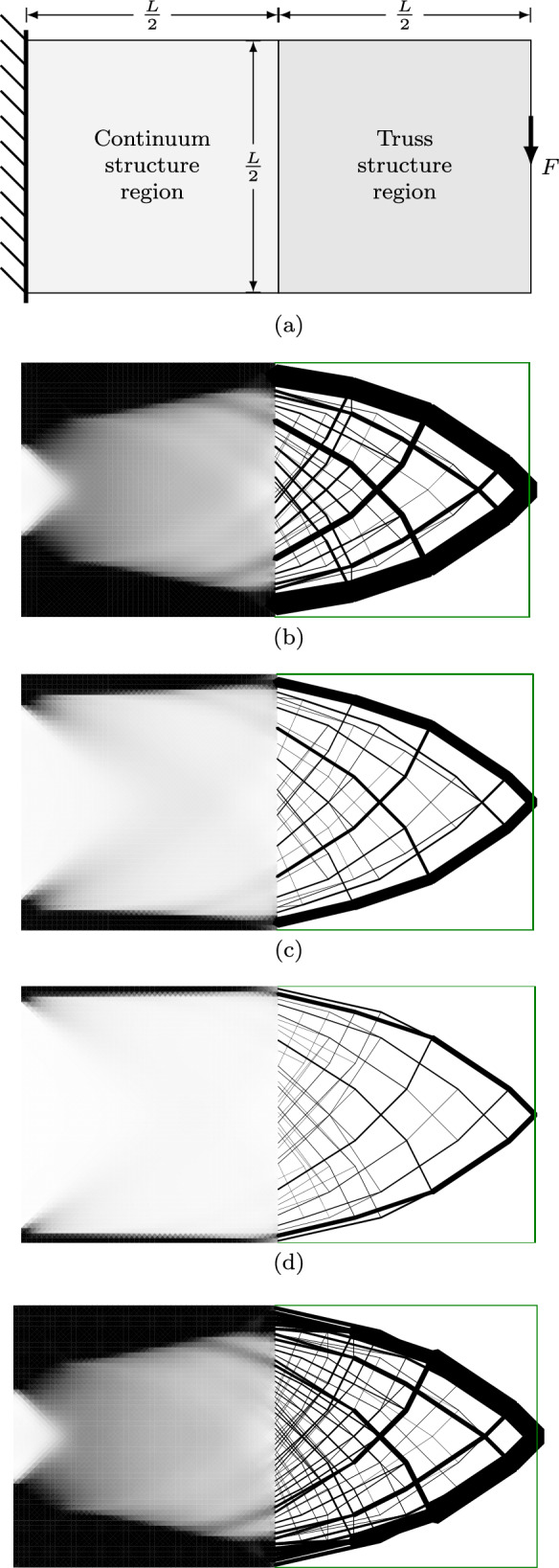
Cantilever with fixed domain division and many-to-many interface: **a** problem specification; **b**–**d** results for different load magnitudes: **b** $F=0.12\sigma L$; **c** $F=0.06 \sigma L$; **d** $F=0.3 \sigma L$

To demonstrate the application of the hybrid optimization problem described in the previous section, a simple cantilever problem is addressed, the geometry of which is described in Fig. 7a; the domain is split into two regions: the left half of the domain is to be filled with continuum material (i.e. a variable thickness plate), whilst the right half is to be filled with a truss structure.

The continuum region is discretized using 10000 continuum elements, a mesh of diagonally quartered squares with edge length $\frac{L}{50}$. The truss region is discretized using a Cartesian grid of nodes with spacing $\frac{L}{10}$, plus additional nodes at the midpoints of each continuum edge along the central interface line. Truss bars connecting to the interface have been permitted to connect to continuum edges within a distance of $\frac{L}{20}$, thus, most truss bars at the interface are connected to five continuum edges.

**Fig. 8:**
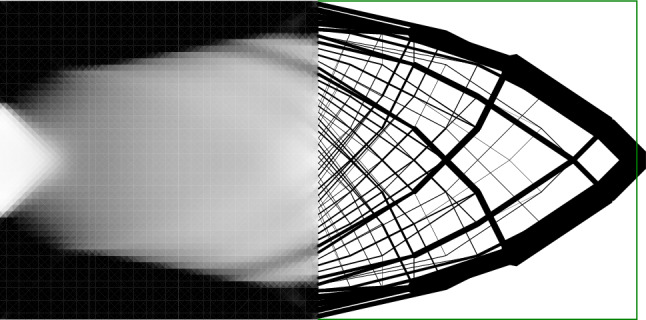
Cantilever with fixed division: result using one-to-many interface with $F=0.12 \sigma L$

The problem has been solved for various magnitudes of the force *F* and the resulting structures are shown in Fig. 7b–d. It can be seen that for larger force magnitudes, the continuum region must extend its solid (black) regions inwards from the boundaries. This also forces the truss bars to connect at nodes inset from the boundaries of the domain. The many-to-many interface is shown to be effective at rationalising the solution in this case; the result using the one-to-many interface in Fig. 8 displays a large number of overlapping bars around the heavily loaded regions.

**Fig. 9:**
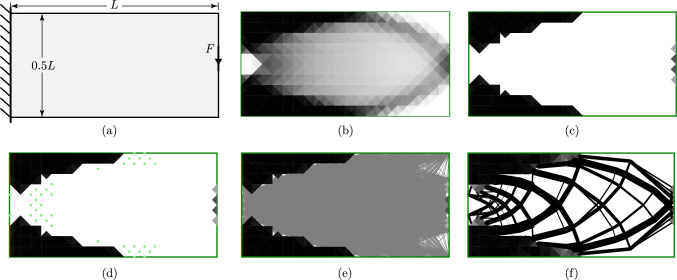
Infill generation procedure example: **a** case description; **b** solve the problem with continuum optimization approach; **c** filter out elements with a density less than the threshold; **d** add additional truss nodes in potentially congested areas; **e** construct truss ground structure; **f** solve the hybrid formulation

At lower force magnitudes, such as in Fig. 7d, the interior of the continuum region is almost white, representing a very low-density region, or a very thin section of a variable thickness sheet. This is unlikely to be practical or desirable in a solution, but the truss forms in these sparse regions are far more interpretable and potentially buildable. Therefore, the next section describes a procedure used to automatically partition the design domain into regions for continuum and truss structures.

## Procedure for infill generation

The combined formulation described in Sect. 2 can be applied to any required division of continuum and truss structure, as demonstrated in Sect. 3. However, for a more automated workflow, it is desirable to combine this with a method that automatically obtains an appropriate division of the domain into continuum and truss regions.

The approach used in this paper is demonstrated graphically in Fig. 9, using a coarse discretization in both the truss and continuum regions for clarity. The following steps are employed:

- Firstly, the design domain, loading and support conditions are defined by the user (Fig. 9a).
- The domain is discretized for the continuum problem, and problem (6) is solved (Fig. 9b).
- Regions of solid structure are identified and retained as the continuum region (Fig. 9c).
- The remainder of the domain is re-discretized with nodes for the truss optimization problem (Fig. 9d).
- The ground structure is generated, with any potential elements which intersect the continuum region removed (Fig. 9e).
- The hybrid problem, (6) or (10), is solved for the combined continuum and truss problem (Fig. 9f).

In this paper, step (c) is implemented by defining solid continuum elements as those where the average value of $\rho$ across the three corners is greater than a threshold, which is taken as 90% of the maximum value. Individual mesh cells that would be surrounded on all sides by the truss region are removed for clarity. Finally, mesh cells that have an applied stress on any edges are kept, to avoid the need to re-define boundary conditions. More complex approaches could be used to interpolate the boundary of the continuum region, but they are beyond the scope of this paper.

In step (d), the truss regions are first discretized using a regular Cartesian grid of nodes at a pre-defined spacing; nodes lying within the continuum regions are removed. To ensure adequate detail can be resolved in the most congested areas of the truss regions, additional nodes are added in regions found to have nearly solid structure in the initial continuum solution (shown as green dots in Fig. 9(d)). Nearly solid structure is here defined as elements where the average value of $\rho$ across the three corners of the element are in the range 80% to 90%. For these cells, the corner point locations are added as additional nodes in the truss problem. Interface nodes are also added along the boundary of the continuum region as described above.

In solving the hybrid problem in step (f), continuum densities are allowed to vary, as in the example in Sect. 3. This provides maximum flexibility, but can sometimes cause small sections of grey structure. Section 6.1 discusses how this can be avoided, and shows that this makes minimal difference to the optimal volumes.

## Examples

This section applies the procedure described above to a number of examples. Firstly, problems common in the literature are addressed (Sects. 5.1, 5.2 and 5.3). Then, in sections 5.4 and 5.5, some problems are presented which demonstrate the efficacy of the proposed approach in particularly challenging scenarios. Throughout, the meshes in the continuum regions are defined using diagonally quartered squares, as visible in the low resolution results in Fig. 9. Note that for visualisation purposes, each continuum element is displayed using the average value of $\rho$ at its three corners, however in the formulation each element has a linearly varying density as described in Kammoun and Smaoui (2014).

Solver times given in the following refer to the time taken by the software (ApS 2020) to solve either (1), (6), (10) or (17) as appropriate. Note that solutions to the hybrid problem rely on the use of a solution for the continuum problem, and so this time should be added to the solver time of the hybrid formulation to give the total requirement.

For illustrative purposes, two renderings of physical models have been created based on optimized hybrid structures. The truss parts of the solid models are created using the nodal expansion approach in Smith et al. (2016). For these results the geometry optimization of (He and Gilbert 2015) has been used to rationalize the truss.

### Cantilever problem

**Fig. 10:**
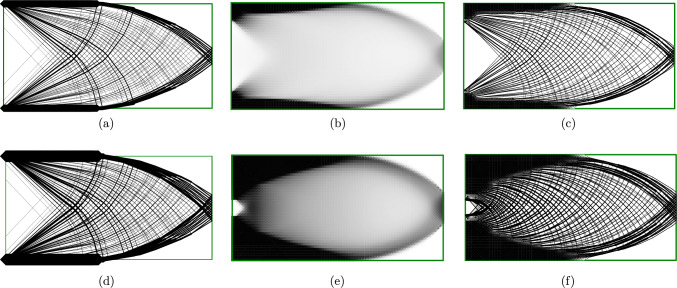
Cantilever problem: **a** truss result with load = 0.040$\sigma L$; **b** continuum result with load = 0.040$\sigma L$; **c** hybrid result with load =0.040$\sigma L$; **d** truss result with load = 0.066$\sigma L$; **e** continuum result with load = 0.066$\sigma L$; **f** hybrid result with load = 0.066$\sigma L$

The cantilever problem in Fig. 9 is studied with a higher resolution in this section. Two sets of results with different loading magnitudes are shown in Fig. 10. For the truss solutions, as the load increases from 0.040$\sigma L$ to 0.066$\sigma L$, the layout of the truss structures remain unchanged and all the member sizes are scaled up by 165%. In Fig. 10(a) and (b), some truss members are located on the top and bottom edges, with their centre lines coinciding with the domain edge. Consequently, half of the members’ volume is located outside the design domain, violating the space constraint. In Fig. 10(a), the space violation is minor but the violation increases as the load magnitude is scaled up, as shown in Fig. 10(d).

Unlike the truss result, the layout of the optimized continuum solution varies as the load magnitude varies. Since the forces are eventually transmitted to the supports, and the elements directly linked to the supports cannot take infinite forces, the solid (black) area at the support boundary expands from the corner positions towards the middle as the load increases. However, since density penalization (Bendsøe 1989) is not used in the continuum approach, the continuum solutions contain many grey elements, violating the ‘black-and-white’ constraint.

By taking advantage of both the discrete and continuum solutions, the hybrid solution satisfies both the space constraint and the ‘black-and-white’ constraint. The corresponding results are shown in Fig. 10c, f. Here and in Fig 9 the one-to-many interface has been used. In these solutions, the continuum region is located near the top and bottom boundary, which is similar to Fig. 10b, e. In addition, the grey areas in Fig. 10b, e are replaced by truss members in Fig. 10c, f.

**Fig. 11:**
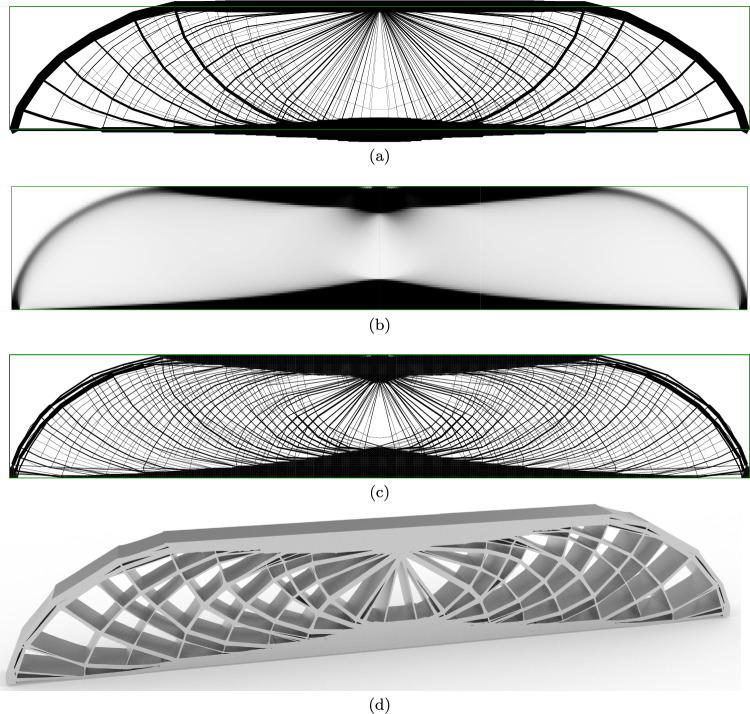
MBB beam: **a** truss solution; **b** continuum solution; **c** hybrid solution (with many-to-many linking and max distance = 0.21); **d** rendered solid model of (**c**)

### MBB beam

Figure 11 shows results for the well-known MBB beam problem. The design domain has a width of *L* and a height of $\frac{L}{6}$. A force of total magnitude *F* is applied at the centre of the top edge of the domain, spread over a total width of $\frac{2}{45}L$ such that the total axial stress applied to the domain is equal to the yield stress $\sigma$. The domain is supported at the outer corners of the base of the domain, with vertical-only support permitted for a distance of $\frac{1}{45}L$ in each corner; i.e. the same total distance as the load is applied over.

In Fig. 11a, the problem is solved using the truss approach, with support points located only in the centre of the region used for the continuum problem. The top and bottom chords in this solution extend outside the design domain. Furthermore, the fan-type regions result in many overlapping elements in the region of the loaded point. Solving the truss-only problems took approximately 7 s.

Figure 11b shows the continuum result, where the top and bottom chords are moved inside the constraints of the domain, thereby reducing the structural depth available and increasing the material required from $2.3195 \frac{FL}{\sigma }$ for the truss solution to $2.48975 \frac{FL}{\sigma }$ for the continuum solution, an increase of 7.3%. However, the areas between the chords are grey and indistinct. Solving the continuum problem took 38 s.

Figure 11c shows the hybrid result with one-to-many interface, preserving the top and bottom chords identified by the continuum problem whilst using truss regions to resolve the detail in the web members. This is able to resolve the fan areas around mid-span, although the continuum region spreads the very centre of the fan to avoid overlapping truss members. The hybrid solution has a volume of $2.5978 \frac{FL}{\sigma }$, and the hybrid conic problem was solved in 491 s (8:11).

Figure 11d shows a solid model of the hybrid MBB beam. For the sake of clarity, a relatively coarse nodal grid is used for this model ($180\times 60$ continuum elements and $37\times 13$ truss nodes are used in Fig. 11c; $45\times 30$ continuum elements and $19\times 7$ truss nodes are used in Fig. 11d).

### Spanning example with point load

**Fig. 12:**
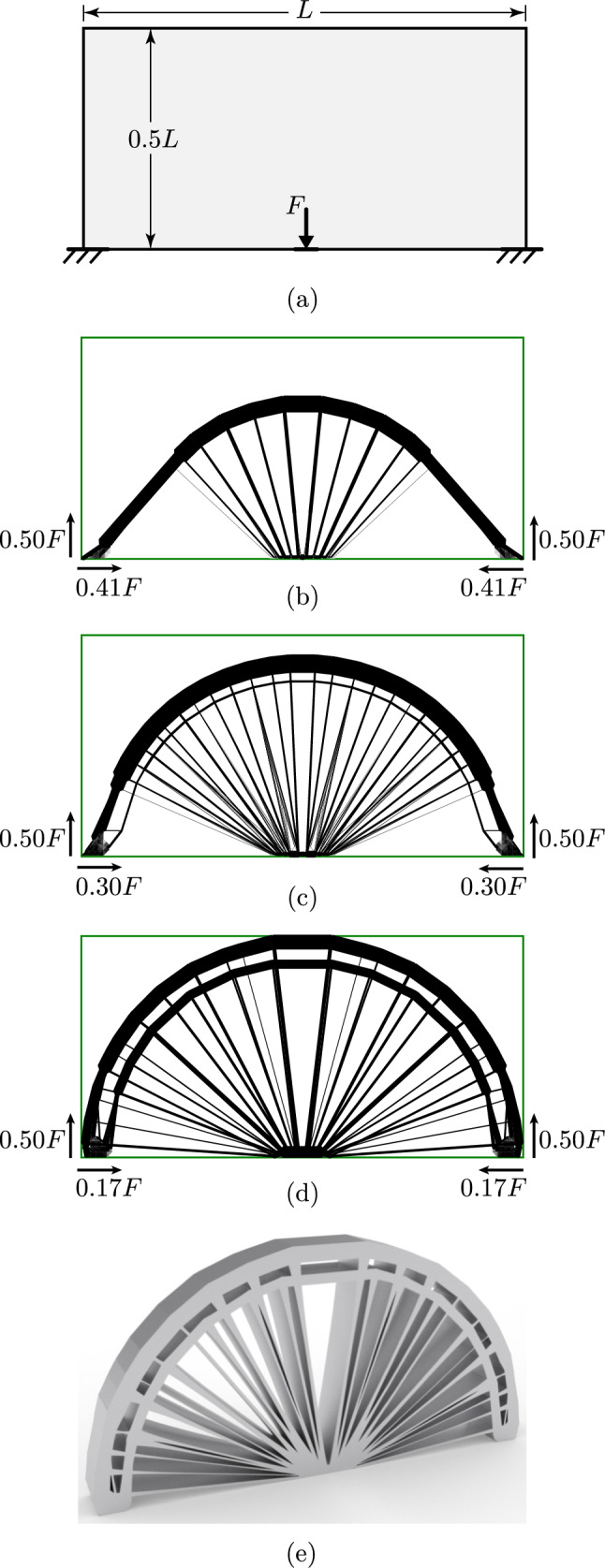
Spanning example: **a** case description; **b** hybrid solution with *F* = 0.060$\sigma L$; **c** hybrid solution with *F* = 0.090 $\sigma L$; **d** hybrid solution with *F* = 0.115$\sigma L$; **e** rendered solid model of (**d**)

Figure 12a shows an example with the supports located at the bottom left and right corners, with loads applied at the centre of the bottom boundary. The results are shown in Fig. 12(b–d) for increasing magnitudes of force *F*. The many-to-many linking is used for this example and for each bar-end *i*, the set ${\mathbb {J}}_i$ contains edges within a distance of 0.04*L*. For the sake of completeness, Fig. 12d has been transformed into a solid model, as shown in Fig. 12e.

At reasonably small loads (Fig. 12b), the structure resembles the well-known truss form for a point load midway between two pinned supports. It contains a central region of radial members and an outer arc, subtending an angle of approximately $90^\circ$. Beyond this, straight lines connect to the supports at an inclination of around $45^\circ$. The variation between the classical truss solution and the solution in Fig. 12b is mainly due to the alteration of the loading from a single point force to distributed loading along the continuum elements.

As the load increases (Fig. 12c,d), the fan structure expands from the middle towards the sides, with the outer rib of the elements becoming almost semi-circular. This result (particularly in Fig. 12d) more closely resembles the truss result for a problem where the supports are vertical only (as in Michell 1904, Figure3).

The reason for this can be understood by considering the continuum material along the supported edges. This material can carry only a limited force, which is controlled by the constraint on the Von Mises stress (5). This effectively limits both the horizontal and vertical reaction forces. At lower loading levels (e.g. Fig. 12b), there is sufficient capacity to carry both the required vertical reactions and horizontal components of similar magnitudes. However, as the load is increased, the required vertical reactions also increase, and there is no longer sufficient capacity to support large horizontal reaction forces. Note that this effect would not be observed in the pure truss solution, where the layout is independent of force magnitudes.

### Cantilever with constriction

**Fig. 13:**
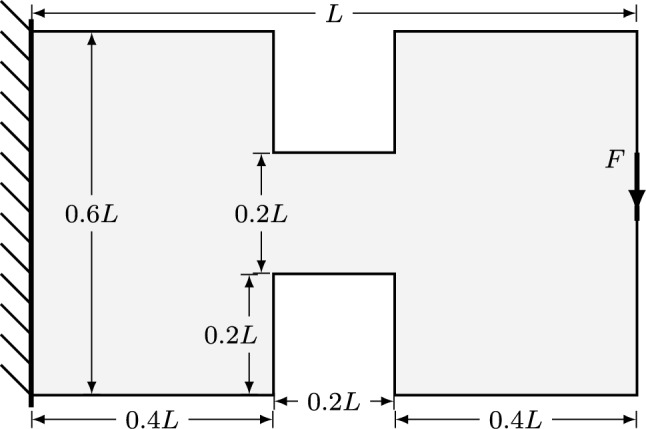
Cantilever with constriction problem: definition

The next example concerns a cantilever, similar to that used in Sect. 5.1. However, in this section the design domain is substantially constrained midway between the load and the supports; the full geometry is shown in Fig. 13.

**Fig. 14:**
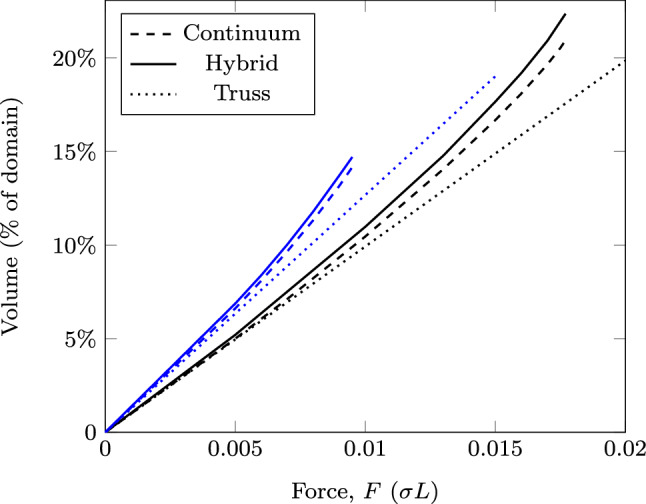
Cantilever with constriction problem: structure volume using different approaches. Blue lines represent solutions for the problem with a reduced height across the whole domain (volume still given as % of the original domain)

**Fig. 15:**
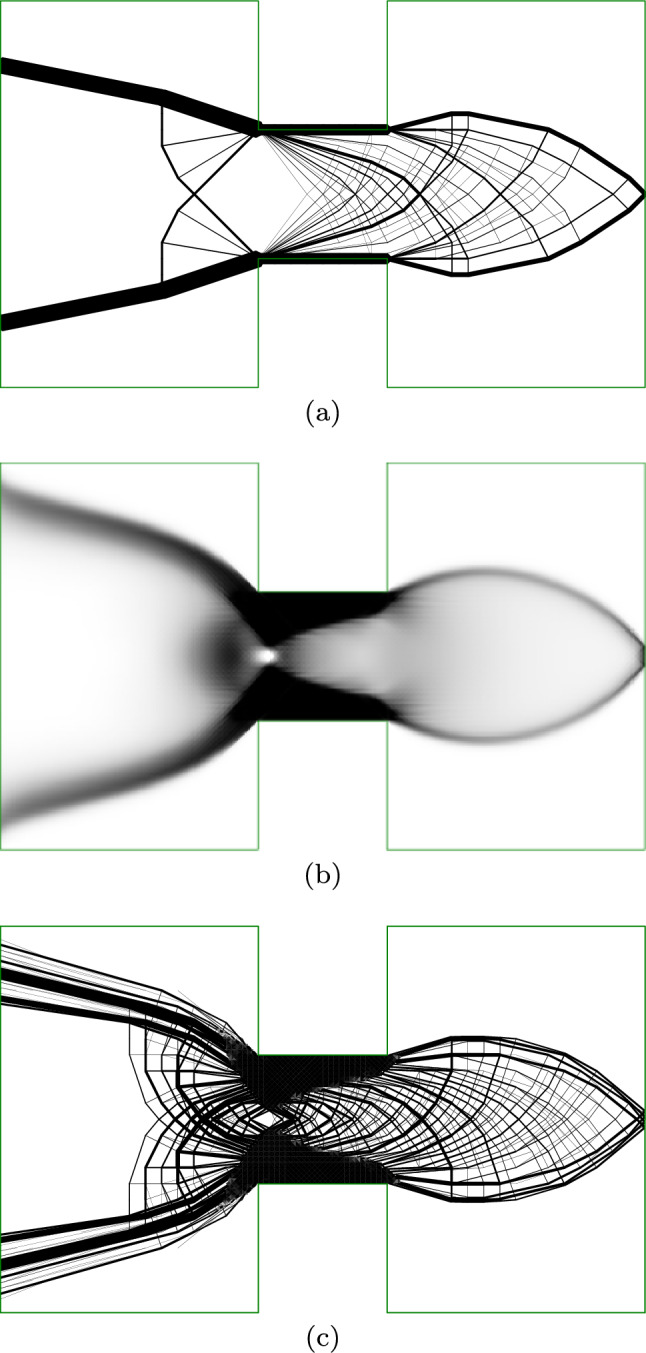
Cantilever with constriction problem—results for $F=0.0175\sigma L$: **a** truss-only result; **b** continuum-only result; **c** hybrid result

**Fig. 16:**
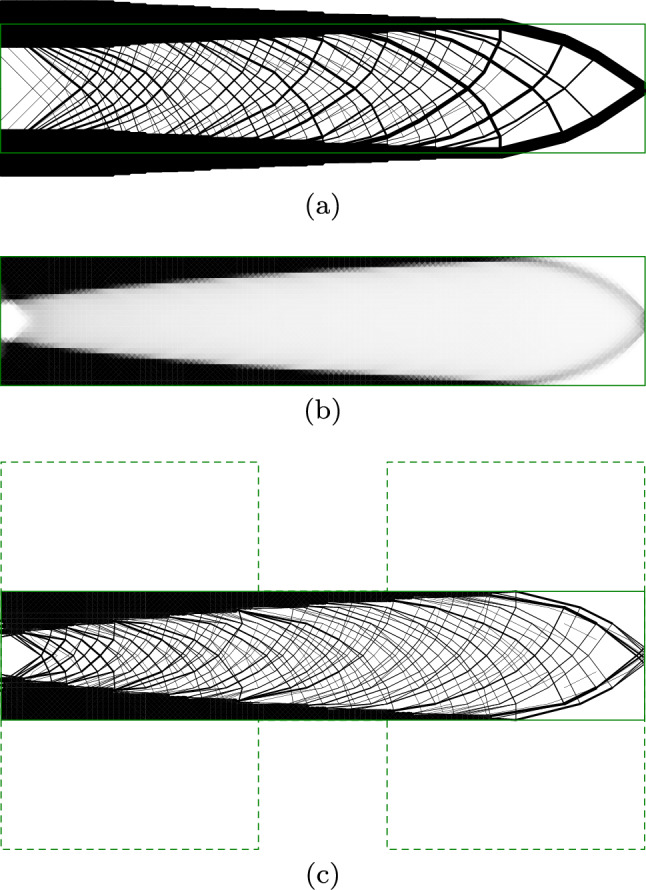
Cantilever with constriction problem - results for $F=0.0095\sigma L$ with fully constricted domain: **a** truss-only result; **b** continuum-only result; **c** hybrid result

The problem has been solved for varying magnitudes of the force *F*, and the volumes of the resulting structures are shown in Fig. 14 using the continuum, truss and hybrid approaches. Figure 14 shows that the volumes of the truss solutions are, as expected, linearly related to the force magnitude *F*. The truss result for $F=0.0175\sigma L$ is shown in Fig. 15a, and other values of *F* give the same layout but with bar thicknesses scaled proportionally. Figure 15a also shows that the bars at the top and bottom of the constriction lie partly outside the design domain; i.e. their centre-lines lie on the boundary line, thus the truss solution does not actually fulfil the requirements of the problem set. The truss-only problem required approximately 3 s to solve.

The volume of the continuum solution lies close to, but slightly above that of the truss solution at low values of *F* (Fig. 14); in this case, the material in the constriction is slightly closer together to ensure it lies strictly within the domain, but at low forces, the overall form is similar to the truss solution. As the force increases, the proportion of the constriction filled with material increases, meaning that any further increase is carried by material much closer to the centre-line of the domain. This compounding effect causes the volume to increase above that predicted by the truss model until, at $F \approx 0.0177\sigma L$, the cross-section close to the left side of the constriction becomes entirely filled with material and forces larger than this cannot be supported. An example solution using the continuum approach for $F = 0.0175\sigma L$ is shown in Fig. 15b; in addition to the significant regions of solid material (black), there are also substantial areas of grey, intermediate density material. The continuum-only problem took around 11 s to solve.

The hybrid solutions have larger volumes than either the continuum or truss solutions (Fig. 14). This is because it combines the restrictions of both of the other approaches to provide the most realistic problem modelling. The example structure for $F=0.175\sigma L$ is shown in Fig. 15c; it shows that the domain constraints are obeyed strictly, with continuum regions present inside the constriction. Furthermore, the indistinct grey regions from the continuum solution are now represented with a clear structure, showing how the forces flow through the form. The presence of continuum regions also mitigates the ‘overlapping members’ problem at the centre of fans: in Fig. 15a there are many overlapping members at the re-entrant corners in the left of the constriction; in 15c, the continuum regions expand to accommodate this. The hybrid problems took between 18 and 25 s to solve, with the more heavily loaded scenarios and their larger continuum regions taking the longest.

In practice, the problem shown in Fig. 13 may be made more amenable to standard designs by limiting the whole domain to the vertical range permitted through the constriction. Figure 14 also shows (in blue) results for such a case using each of the three approaches. From the truss-only problems, reducing the design domain in this way implies an increase in volume of 28%. However, the truss forms, again, significantly violate the domain constraints, as shown in Fig. 16a. The continuum and hybrid results further increase the volume of the optimal structure in order to ensure it lies wholly within the domain, eventually leading to a maximum load for this problem of just over $0.0095\sigma L$. At $F= 0.0095\sigma L$, the reduction in the design domain actually implies an increase in volume of 42%.

**Fig. 17:**
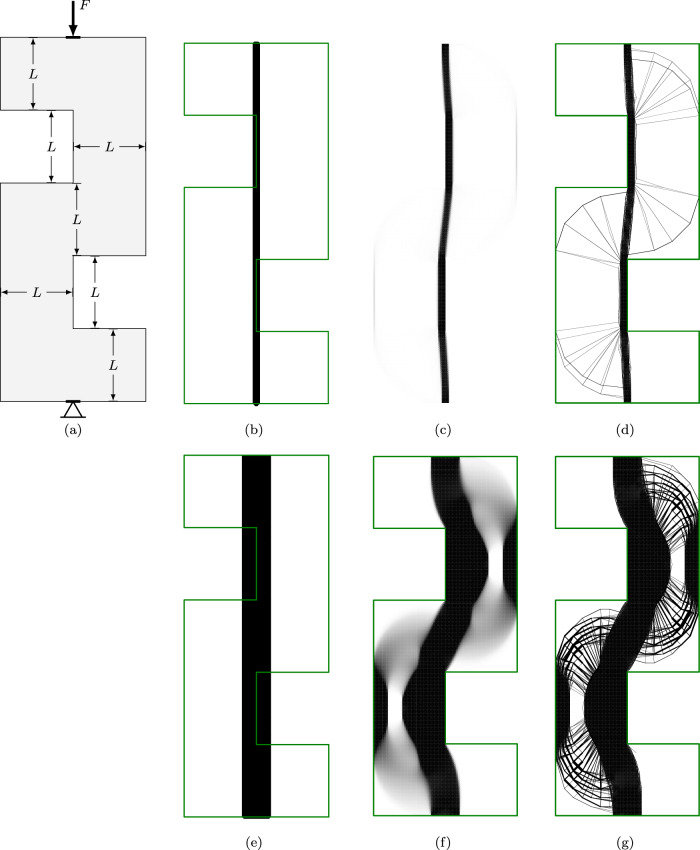
Tower example: **a** problem definition; **b**–**d** optimal forms with $F=0.1 \sigma L$; **e**–**g** optimal forms with $F=0.4 \sigma L$: **b** and **e** truss approach with single point load; **c** and **f** continuum approach; **d** and **g** hybrid approach. The domain boundary has been removed from (**c**) to better show the outlying areas of the structure

**Fig. 18:**
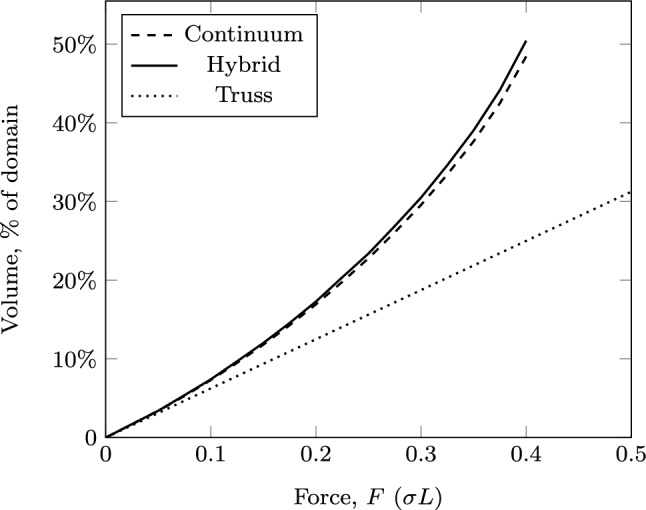
Tower example: structure volume using different approaches

### Tower

This example involves the simple transmission of a vertical force, *F*, directed towards a line of support. The domain is shown in Fig. 17a, where there are forbidden regions intruding up to the line of action of the force, but not beyond. Thus, a simple truss model results in the force being transmitted through a bar whose centre-line lies along the boundary of the domain; i.e. half of the width of the bar is outside the permitted domain (Fig. 17b,e). To correct this, the bar would need to be moved, leading to eccentricity of the loading and thus generating additional moments.

To consider the problem using the continuum approach, the point loading and supports are both distributed over the same short width $\frac{\sigma }{F}$, chosen such that the stress imposed by the force is equal to the yield stress $\sigma$. Note that merely spreading the force in this manner does not ameliorate the problems with the truss results, as the forces are simply brought back together using smaller members converging at the top of the first constriction, where they join to form a larger member identical to the ones in Fig. 17b, e.

The continuum results in Fig. 17d, h show the central column being correctly deviated to remain wholly within the design domain. To support the moments resulting from this eccentricity, additional support structure is required. However, this additional structure is very fine compared to the central column especially when the imposed force is small; for example, in Fig. 17c much of the supporting structure cannot be visibly distinguished from void, leading to the appearance of floating areas of material.

By using the hybrid approach proposed here, both of these issues can be dealt with. The continuum stage first ensures that the central column is entirely within the design domain, and then the truss representation allows for a useful depiction of the finer support structure. The hybrid solutions use a continuum mesh with 0.025*L* spacing and a truss nodal grid at 0.125*L* spacing, the many-to-many interface is used, and for each bar-end *i*, the set ${\mathbb {J}}_i$ contains edges within a distance of 0.03*L*. At lower loading levels, the continuum region is maintained only along the central column line, whilst at larger loading levels, continuum regions are also required on the outside of the domain as part of the supporting structure.

For the results shown in Figs. 17 and 18, the time required to solve the truss-only problems was up to three seconds, whilst the continuum problems took between 40 and 50 s. The hybrid problems took between 98 and 221 s to solve, with longer times required for the more heavily loaded structures, where a larger proportion of the continuum region is maintained.

## Discussion and outlook

This paper has presented an approach to combine both truss and continuum parametrisations in order to address optimization problems with both high and low volume fraction regions. Here, the formulation has been derived and tested based on a single type of problem – limit design under a single loading case. Nonetheless, the basic principle of combining truss and continuum approaches is one which could be explored in a wide range of scenarios. This section will discuss where there is scope for variation within the framework and suggest avenues for future work.

### Variations to the optimization problem

In this paper, the continuum density variables $\rho$ have been allowed to vary whilst solving the hybrid problems, as for the continuum-only problem. However, this does mean that there is the possibility that some grey elements may persist in the final solutions.

**Fig. 19:**
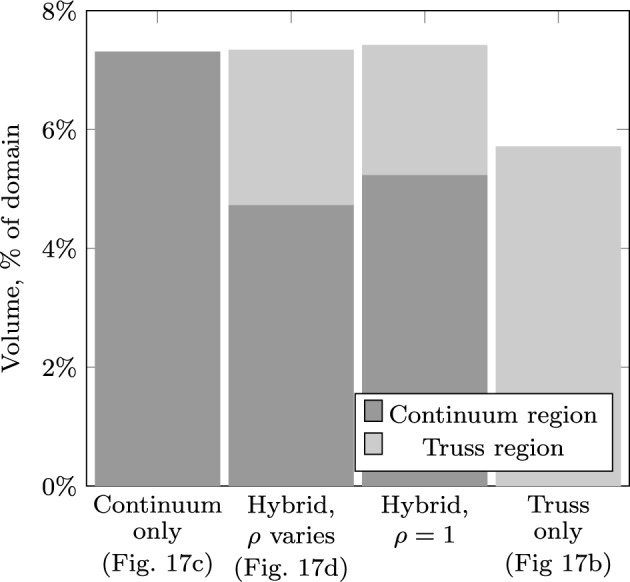
Tower example: breakdown of volume within continuum and truss regions for case $F=0.1\sigma L$, highlighting the impact of solving with fixed continuum densities $\rho$ in the hybrid problem

A simple approach to counter this possibility is to fix the values of $\rho$ to 1 in the hybrid stage. Across the hybrid examples presented herein (Figs 10, 11, 12, 15,  16, 17) this makes less than 0.5% difference in the optimal volume for all examples except that shown in Fig. 17d, where the volume increase is 1.1%; This example is shown in more detail in Fig. 19.

In Fig. 19, fixing $\rho = 1$ increases the volume of the continuum regions by 6.9%, but also allows the volume in the truss region to reduce by 5.8%, giving the overall change of around 1.1%. This therefore suggests that there are regions where either a truss or continuum load-path would give very similar material usage. Generally, the issue of grey elements is seen around the boundaries of the continuum regions (see also Fig. 10c/f) which further supports this conclusion.

The reason why this example should be particularly affected can be understood by considering the continuum solution in Fig. 17c. This shows sections where the central tower should be close to (but not exactly) vertical. This boundary cannot be well represented by the vertical and $45^o$ edges in the continuum mesh used here, and so the boundary in the hybrid problem is quite irregular. Therefore neither the truss or continuum region is fully capable of containing the required load-path. This is further supported by the form of Fig. 17d, where a large number of truss elements run alongside the continuum region, especially in the non-vertical parts.

Based on this, it is likely that a more sophisticated approach to dividing the continuum and truss regions would be a promising avenue for further investigation. For example, this could be approached by re-meshing to ensure smoother boundaries between the truss and continuum regions when converting the continuum result to hybrid problem, or by allowing the boundary between regions to be refined as part of a shape/geometry optimization stage.

In both the continuum and truss optimization fields it is common to refine the structures obtained from topology optimization via geometry or shape optimization stages. The rendered figures presented here (Fig. 11d and 12e) partially implement this via the approach of He and Gilbert (2015). However, in those cases only nodes entirely in the truss domain (not on the interface) were permitted to move, thus requiring no particular modifications to the approach of He and Gilbert (2015).

To apply shape optimization to the continuum part of the structure, there are a huge range of possible approaches which have been developed. A review of available methods may be found in Upadhyay et al. (2021). Of these, parameter-free approaches seem most attractive for this task due to the conceptual similarity with truss geometry optimization approaches and the lack of manual reparametrization required. However, shape optimization approaches have not yet been applied in the context of plastic limit analysis, and it is recommended that such approaches should be tested on the continuum problems first for simplicity before being applied within the hybrid formulation proposed here.

The final consideration in considering shape optimization of these hybrid structures is how to address the interface between the continuum and hybrid regions. Various possibilities exist, such as directly linking continuum nodes to the relevant end-points, or adopting concepts from the MMC approaches to project truss elements into continuum meshes. Further work will be required to assess the efficacy and computational efficiency of these possibilities.

As an alternative to post-processing the hybrid solution, it may be possible to incorporate de-homogenization principles to immediately generate preferable nodal locations in the truss region; for example, by applying the principles of Larsen et al. (2018). By maintaining an explicit continuum parametrisation in heavily loaded regions, the interventions required to, e.g. create nodes at fan centres and the possibility of elements extending outside of the domain would be negated.

### Extension to a wider range of structural problems

Here, single load-case formulations have been considered for simplicity. However, the formulations presented here can be easily extended to multiple load-case problems. The required modifications to the plastic limit analysis continuum formulation can be found in Kammoun (2016), and essentially require that the stress variables and equilibrium/yield constraints are duplicated for each case, whilst the density variables are common to all cases. Similarly, in the truss formulation, multiple load cases are addressed by defining the force variables and equilibrium/yield constraints separately for each load case, with the area variables common across all cases Dorn et al. (1964).

Extension of the hybrid formulation here would follow a similar pattern. In the one-to-many interface problem (10), the only changes required would be as in the existing formulations; to define separate continuum stress/truss force variables for each load-case, coupled by common density/area variables, and to impose the equilibrium and yield constraints for each load-case. For the many-to-many interface problem, (17), an additional change would be required; the direct use of the component forces, ${\hat{q}}_{i,d}$ in the objective function would need to be replaced with corresponding component area variables to correctly combine the forces from different cases.

When multiple load cases are present, the optimal elastic and plastic designs diverge. Therefore it may become attractive to consider compliance optimization (as is more common within the continuum optimization field), rather than the plastic optimization here (which is more common in the truss optimization field). Either or both may correspond to the design requirements in a real scenario.

For compliance optimization, conic formulations are also available for both the truss and continuum problems (Makrodimopoulos et al. 2010), and therefore implementation of the a hybrid approach would follow similar principles to that laid out here. The biggest difference in implementation will depend on whether the continuum formulation used requires forces to be applied on edges (as in the plastic formulation used here) or at nodes (more common in compliance optimization problems). Nonetheless, particularly when the many-to-many interface is used, this difference essentially amounts only to altering the point of action for the component forces (Fig. 5b).

Here, the heuristic division between continuum and truss regions has led to relatively fine truss elements being present in the examples considered in Sect. 5. Even when the one-to-many interface was employed, the truss elements are still rather slender and therefore a truss model is likely to be applicable. If the many-to-many formulation was applied in scenarios where thicker elements exist in the truss section (e.g. Fig. 7), it may be necessary to consider whether the elements in the truss region would be better modelled as beam elements. The modelling of beam elements is challenging within the ground structure framework as it generally results in a non-convex problem, although some approximations are possible to mitigate this (Lu et al. 2025). There may also be scope to adopt this paradigm for pure bending-type problems, e.g. combining continuum shells with discrete grillage elements.

### Comparison with other available approaches

The approach proposed here achieves similar aims to those of other methods which aim to reconstruct detailed black-and-white structures from low resolution continuous solutions. The closest analogy is to de-homogenization, although some comparisons with other multi-scale approaches are also possible (Wu et al. 2021). As with de-homogenization, the structures produced here are generally seen to display a moderate range of length scales (as opposed to the wildly different length scales exhibited in, e.g. Rank-N laminates). This variation in length scale is wide enough to permit much greater detail than is practical to obtain via direct density-based methods, whilst still falling within what is reasonably manufacturable. In this subsection, comparisons between this approach and other multi-scale strategies will be briefly reviewed.

The principal advantage of explicitly using a truss approach to parametrize low-density regions is in removing issues relating to ensuring connectivity and re-interpreting locations where there are multiple possible microstructures which can represent the required properties. In particular, de-homogenization methods can struggle when there are sudden changes in orientation or density of the optimal lines of force – a truss-based approach has no such limitations. Also, de-homogenization requires that different microstructures are defined depending on, e.g. whether the problem has just one or multiple load cases, whereas the truss-based approach can freely construct the microstructure form from the individual elements.

The main drawbacks of using a truss-based approach mirror those of the stand-alone truss formulation. For example, possible overlapping or gaps between elements at connections, or difficulties with creating pinned connections during manufacturing. It is notable that both of these issues are lessened when the thickness of elements is reduced, e.g. when the truss region is located in a region with low volume fraction. A minor issue is related to explicit control of length scales in the final structure, in the formulation presented here it is not possible to explicitly specify a minimum feature size, although this can be indirectly addressed by altering the nodal discretization (where finer discretizations generally lead to a greater number of thinner elements).

At the time of writing, multi-scale topology optimization is much more mature than the approach we propose here and has been adapted to a wider range of scenario types. Nonetheless, it is worthwhile to point out that most additional structural considerations which have a viable treatment in both the continuum and ground structure fields individually, will likely be amenable to adaptation in the manner presented here.

## Conclusions

The hybrid approach presented can overcome the limitations observed in continuum and truss-based optimization approaches, respectively. Specifically, the approach has been shown to mitigate the existence of truss bars that extend outside of the domain due to their thickness, and of congested regions caused by converging truss bars. Meanwhile, clarity is improved through the use of trusses in areas of sparse structure, where continuum approaches would produce indiscernible areas of pale grey.

The interface required to connect the continuum and hybrid regions maintains the convexity of the ground structure formulation and the variable thickness sheet continuum approaches. This allows globally optimal solutions to be obtained for a given division of continuum and truss regions. To achieve this for cases where truss bars may become larger than a single continuum element, some approximations are required, with the effect of these limited by a user-defined distance.

To obtain a suitable division of truss and continuum regions, a simple two-stage heuristic has been demonstrated. This requires only a small number of easily interpretable parameters to be specified, principally the cut-off values for solid and congested (additional truss node) regions.

As the approach requires only two stages, the computational effort required is relatively low, particularly compared to the iterative approaches often used to drive continuum topology optimization results to pure solid/void solutions. Nonetheless, the computational effort required is significantly greater for the hybrid approach compared to the pure truss solution.

Overall, this approach provides a conceptually simple and computationally efficient approach to generate optimal structural designs where both fine detail and free-form heavily loaded regions are required.

## Appendix

This appendix provides details of the values for the matrices required in the formulations used herein.

In the continuum approach of Kammoun and Smaoui (2014). Stresses vary linearly within an element, thus the normal and shear stresses can be represented as

18 $$\begin{aligned} \sigma _x = \sum _{i=1}^3N_i\sigma _{xi};\quad \sigma _y = \sum _{i=1}^3N_i\sigma _{yi};\quad \tau _{xy} = \sum _{i=1}^3N_i\tau _{xyi} \end{aligned}$$

where $N_i$ are linear shape functions given by

19 $$\begin{aligned} N_i= & (\xi _i+\eta _ix+ \zeta _iy)/2A \end{aligned}$$

20 $$\begin{aligned} \xi _1= & x_2y_3-x_3y_2; \; \eta _1 = y_2 - y_3; \; \zeta _1 = x_3 - x_2 \end{aligned}$$

21 $$\begin{aligned} \xi _2= & x_3y_1-x_1y_3; \; \eta _2 = y_3 - y_1; \; \zeta _2 = x_1 - x_3 \end{aligned}$$

22 $$\begin{aligned} \xi _3= & x_1y_2-x_2y_1; \; \eta _3 = y_1 - y_2; \; \zeta _3 = x_2 - x_1 \end{aligned}$$

23 $$\begin{aligned} A= & |\eta _1\zeta _2 - \eta _2\zeta _1| \end{aligned}$$

As already stated in Sect. 2.2, three types of equilibrium constraints are used in the continuum optimization formulation. The details of the transformation matrices and the stress vectors can be found as follows.

For the continuum element constraint, the shape function matrix ${\textbf{N}}_e$ is defined for each element *e*.

24 $$\begin{aligned} {\textbf{N}}_e= & \begin{bmatrix} \eta _1 & 0 & \zeta _1 & \eta _2 & 0 & \zeta _2 & \eta _3 & 0 & \zeta _3\\ 0 & \zeta _1 & \eta _1 & 0 & \zeta _2 & \eta _2 & 0 & \zeta _3 & \eta _3 \end{bmatrix} \end{aligned}$$

25 $$\begin{aligned} {\varvec{\sigma }}_e= & \Big [\sigma _{x1}, \sigma _{y1},\tau _{xy1}, \sigma _{x2}, \sigma _{y2}, \tau _{xy2}, \sigma _{x3}, \sigma _{y3}, \tau _{xy3} \Big ] \end{aligned}$$

To construct the edge constraints (both internal to the continuum mesh and those lying on its boundary) a transformation matrix, ${\textbf{A}}_d$ is defined based on the angle $\theta _d$, the angle between edge *i* and the positive *x*-axis.

26 $$\begin{aligned} {\textbf{A}}_d= & \begin{bmatrix} {\textbf{T}}_d & 0 \\ 0 & {\textbf{T}}_d \end{bmatrix} \end{aligned}$$

27 $$\begin{aligned} {\textbf{T}}_d= & \begin{bmatrix} \sin ^2\theta _d & \cos ^2\theta _d & -\sin 2\theta _d \\ -\frac{1}{2}\sin 2\theta _d & \frac{1}{2}\sin 2\theta _d & \cos 2\theta _d \end{bmatrix} \end{aligned}$$

For boundary edges, the internal stresses are defined at the end-points 1 and 2 of the edge.

28 $$\begin{aligned} {\varvec{\sigma }}_{d} = \big [ \sigma _{x1}, \sigma _{y1} ,\tau _{xy1} , \sigma _{x2} , \sigma _{y2}, \tau _{xy2} \big ]^\textrm{T} \end{aligned}$$

The external tractions (normal and tangent to the edge) are also defined at points 1 and 2, and are found in the matrix ${\textbf{t}}_d$:

29 $$\begin{aligned} \begin{aligned} {\textbf{t}}_d = {\Big [t_{n,1}, t_{s,1}, t_{n,2}, t_{s,2} \Big ]} \end{aligned} \end{aligned}$$

where *q* describes the applied stress normal to the edge, and *t* describes the applied shear stress.

Meanwhile, for edges within the continuum mesh, the internal stresses are defined at each end and within each of the two connected elements. These points are numbered as shown in Fig. 2, and assembled into separate vectors for each of the two elements:

30 $$\begin{aligned} {\varvec{\sigma }}_{d, \textrm{I}}&= \big [ \sigma _{x1}, \sigma _{y1} ,\tau _{xy1} , \sigma _{x2} , \sigma _{y2}, \tau _{xy2} \big ]^\textrm{T} \end{aligned}$$

31 $$\begin{aligned} {\varvec{\sigma }}_{d, \mathrm{I\hspace{-0.2ex}I}}&= \big [ \sigma _{x3}, \sigma _{y3} ,\tau _{xy3} , \sigma _{x4} , \sigma _{y4}, \tau _{xy4} \big ]^\textrm{T} \end{aligned}$$

## Declarations

### Replication of results

All required details of the formulation and examples presented are provided in the manuscript. Additional details are available on reasonable request to the authors.
